# Asymmetric Isolation and the Evolution of Behaviors Influencing Dispersal: Rheotaxis of Guppies above Waterfalls

**DOI:** 10.3390/genes11020180

**Published:** 2020-02-09

**Authors:** Léa Blondel, Sandra Klemet-N’Guessan, Marilyn E. Scott, Andrew P. Hendry

**Affiliations:** 1 Redpath Museum and Department of Biology, McGill University, 859 Sherbrooke St. W., Montréal, QC H3A 0C4, Canada; andrew.hendry@mcgill.ca; 2 Department of Biology, Trent University, 2140 East Bank Drive, Peterborough, ON K9L 1Z8, Canada; sandraklemet@trentu.ca; 3 Institute of Parasitology, McGill University (Macdonald Campus), 21,111 Lakeshore Drive, Ste. Anne-de-Bellevue, QC H9X 3V9, Canada; marilyn.scott@mcgill.ca

**Keywords:** rheotaxic behavior, introduced populations, dispersal, guppies, waterfalls

## Abstract

Populations that are asymmetrically isolated, such as above waterfalls, can sometimes export emigrants in a direction from which they do not receive immigrants, and thus provide an excellent opportunity to study the evolution of dispersal traits. We investigated the rheotaxis of guppies above barrier waterfalls in the Aripo and Turure rivers in Trinidad—the later having been introduced in 1957 from a below-waterfall population in another drainage. We predicted that, as a result of strong selection against downstream emigration, both of these above-waterfall populations should show strong positive rheotaxis. Matching these expectations, both populations expressed high levels of positive rheotaxis, possibly reflecting contemporary (rapid) evolution in the introduced Turure population. However, the two populations used different behaviors to achieve the same performance of strong positive rheotaxis, as has been predicted in the case of multiple potential evolutionary solutions to the same functional challenge (i.e., “many-to-one mapping”). By contrast, we did not find any difference in rheotactic behavior above versus below waterfalls on a small scale within either river, suggesting constraints on adaptive divergence on such scales.

## 1. Introduction

Populations that become newly established in isolated places, such as on islands or above waterfalls or dams, provide excellent opportunities to study evolutionary processes [1,2,3]. The establishment of these populations can reflect natural processes or human activities. Relevant natural processes include rare climatological events (e.g., storms or floods) or geological events (e.g., earthquakes or volcanoes) that spread organisms to new places [4]. Relevant human actions can reflect unintentional introductions (e.g., rats on ships or zooplankton in ballast water) or intentional releases (e.g., biocontrol or “assisted migration”; [5]). Human-driven establishment can also happen when scientists introduce organisms into new environments to test ecological or evolutionary theories [6,7,8,9]. Especially in these latter cases, a particular emphasis is placed on maintaining strong isolation of the new populations, such that evolutionary and ecological dynamics can unfold without the complicating influence of unplanned inputs from external sources [8,10,11]. 

In a number of these establishments, regardless of their cause, the resulting isolation of new populations is asymmetrical: that is, they do not receive gene flow from other populations whereas they can still export genes to at least some other populations. For instance, fish establishing in headwater lakes or above waterfalls are unlikely to receive many immigrants from further downstream, yet they commonly export emigrants into downstream populations [12,13,14]. This unidirectional export of genetic material from asymmetrically isolated populations into non-isolated populations can have considerable neutral (altered genetic diversity) or adaptive (altered adaptive potential) consequences [15]. In particular, the phenotypic traits of newly established populations might be quite different from nearby non-isolated populations into which they export alleles. Accordingly, some studies have shown that introduced populations established high in stream watersheds can have massive genetic consequences for natural populations further downstream [6,12,16]. However, if the tendency for emigration has a (at least partial) genetic basis, alleles that increase emigration will be lost from the populations and—owing to asymmetric isolation—will not return [17]. Hence, asymmetrically isolated populations should evolve traits that reduce emigration and, consequently, their effects on downstream populations should decrease through time. 

In riverine fishes, one way to avoid downstream emigration is to increase the tendency to maintain position in the stream and swim against the current, referred to as positive rheotaxis [18,19,20]. Rheotactic behavior is genetically based in many fishes [21,22,23], and so we hypothesize that asymmetrically isolated riverine fish populations (those above waterfalls) should evolve positive rheotaxis. Consistent with this hypothesis, several studies have shown that fish populations above waterfalls have higher positive rheotaxis than those below waterfalls [22,23,24]. Few studies, however, have asked how repeatable this evolution might be, which is an inference that requires assaying multiple populations of a focal species that independently established above waterfalls. Therefore, our first objective was to test whether two different above-waterfall populations of Trinidadian guppies (*Poecilia reticulata*) show positive rheotaxis. 

Positive rheotaxis could be attained in several different ways. For instance, fish could avoid high current, could increase their upstream orientation, or could swim faster or longer in the upstream direction. Positive rheotaxis is therefore a “performance” metric that could be achieved through multiple different behavioral, physiological, or morphological solutions. Thus, the problem of evolving positive rheotaxis in above-waterfall populations is another example of many-to-one mapping [25]. In such cases, a key question becomes whether parallel evolution of performance (positive rheotaxis) is the result of non-parallel solutions. Therefore, our second objective was to ask whether the two above-waterfall guppy populations evolved positive rheotaxis through the same or different behavioral solutions. 

We also wanted to consider the possibility of fine-scale variation in rheotaxis. In particular, we hypothesized that increasing degrees of asymmetric isolation should lead to increasing selection against behaviors favoring downstream emigration. For example, the fish above a series of barrier waterfalls might be expected to show stronger positive rheotaxis than the fish between those waterfalls, which might be expected to show stronger positive rheotaxis than the fish below those waterfalls. Thus, our final objective was to evaluate these hypotheses by testing the rheotaxis of guppies from above, below, and between two barrier waterfalls in each river.

To our knowledge, the rate at which rheotaxis evolves has never be considered. Given that contemporary (or “rapid”) evolution has been documented in many organisms experiencing strong selection [2,26,27], especially guppies [28], we suggest that positive rheotaxis will evolve quickly in guppies introduced above waterfalls. One of our two study populations was introduced by scientists, which has given us the irresistible temptation to speculate on the potential rapidity of rheotaxis evolution. (We acknowledge here that this speculation was discouraged by anonymous reviewers.) However, given the impossibility of evaluating initial rheotaxis in the introduced population, and given ambiguity as to the precise ancestral source (details below), our inferences on this point will remain speculative. 

## 2. Materials and Methods 

### 2.1. History of an Introduction and Its Effects

In 1957, guppies were introduced by Caryl Haskins from below all barrier waterfalls in the Guanapo River (Caroni drainage) into a previously guppy-free site above a series of barrier waterfalls in the Turure River (Oropuche drainage) [29]. Uncertainty exists as to the exact source population for this introduction, with suggestions including the Arima River [6,30,31,32], the Guanapo River [15,29], or the Aripo River [33]. This introduction was unknown to scientists until Shaw et al. (1991) found a puzzling signature of Caroni genotypes in the lower Turure River of the Oropuche drainage. They formulated an hypothesis that Caroni drainage guppies had been introduced into the Turure River, which was later confirmed by personal communication from Caryl Haskins [30]. Guppies in the Caroni and Oropuche drainages had otherwise been isolated from each other for approximately 0.6–1.2 million years, thus generating dramatic genetic differences [30,34,35]. After the introduction, however, a strong genetic signature in both nuclear and mitochondrial genes of Guanapo fish was detected well downstream of the initial asymmetrically isolated introduction site in the Turure River [15,31,33]. For instance, Shaw et al. (1992) investigated six enzyme-coding loci and found that Turure fish located 1 km downstream of the introduction site had alleles normally found only in the Caroni drainage [31]. Becher et al. (2000) investigated mitochondrial DNA and found that only 12% of genotypes in the downstream sites corresponded to the native Oropuche population. Finally, Fitzpatrick et al. (2015) investigated microsatellite markers and found that sites located 1 km downstream of the introduction primarily clustered with introduced fish and not with the native population [15]. In short, the fish that Haskins first introduced initially had very strong downstream genetic effects reflecting substantial emigration and, presumably, the absence of strong positive rheotaxis.

### 2.2. Fish Sampling

**Figure 1:**
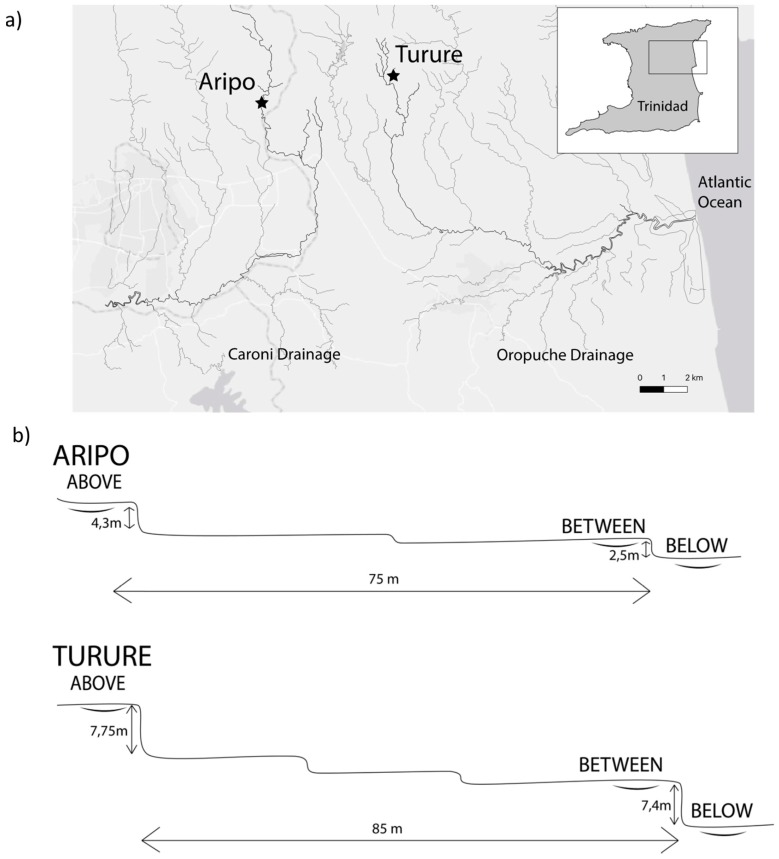
(**a**) Location of collection sites in the two rivers in the Northern Mountain range in Trinidad. (**b**) Profile for the two rivers showing the relative location and height of waterfalls in relation to the distance between them. Rivers flow from the left (upstream) to the right (downstream). Above, Between and Below represent the three collection sites per stream.

We used butterfly nets to collect guppies during the dry season (March 2015) from two rivers: the Aripo River in the Caroni drainage and the Turure River in the Oropuche drainage (Figure 1). The collection sites in both rivers are “Low Predation” (LP) owing to the absence of predatory fish other than *Rivulus hartii* [36]. Established guppy populations were present downstream of the collection area in both rivers and were found upstream of the collection area in the Aripo but not the Turure. With the guppies being asymmetrically isolated in both sampling locations by downstream waterfalls, it allowed us to test Objective 1 (both populations should show positive rheotaxis) and Objective 2 (whether positive rheotaxis was achieved through similar behavioral strategies in both populations). In each of the two rivers, collections took place from three pool types: one pool above an upstream waterfall (“Above”), one pool below a downstream waterfall (“Below”), and one pool between the two waterfalls (“Between”) (Figure 1). This sampling allowed us to test Objective 3: whether rheotaxis showed fine-scale variation depending on their location relative to multiple barrier waterfalls. Collected fish were immediately transferred to a field lab in Trinidad, where they were kept in separate tanks according to their pool type/river for one week prior to transport to Montréal (Québec, Canada) by air cargo. 

Fish were then kept in the laboratory after an 8-month quarantine and lab acclimation period. They were separated in river and pool type specific tanks in still water with an air pump and were fed daily with brine shrimp. The experiments used a combination of the wild-caught females (F0) and their female offspring (F1), which were raised in common-garden conditions. The experiments used females rather than males because female guppies generally express high site fidelity, contrary to male guppies that show more movement between pools [37]. Hence, wild-caught males might have been less representative of the local population, perhaps having recently arrived from elsewhere.

### 2.3. Rheotaxis Apparatus

**Figure 2:**
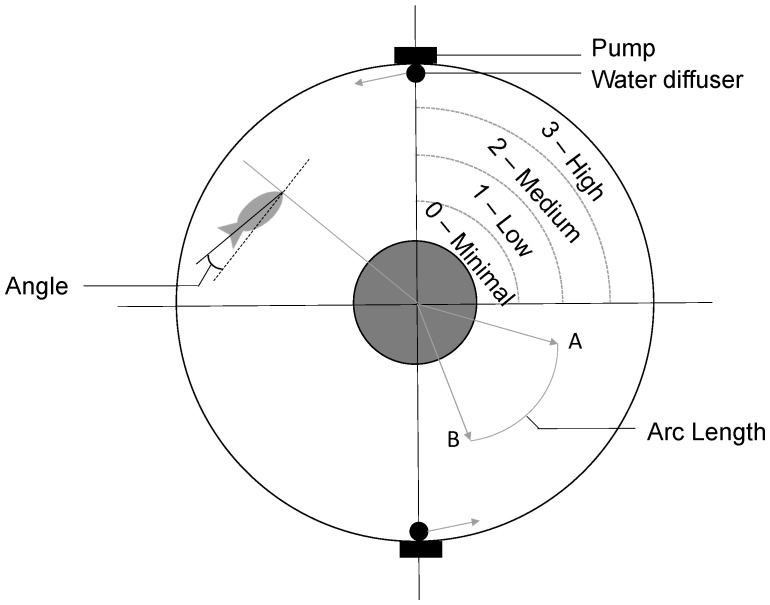
The experimental circular-flow tank. Dotted lines delineate different approximate flow regimes (High, Medium, Low and Minimal: upper right quadrant). Distance was measured as the arc length distance between two points (lower right quadrant). Angle was measured as the alignment of the fish against the flow (upper left quadrant). Direction of the water is given by arrows from the water diffusers. The tank dimensions were: testing chamber diameter = 65 cm; inside diameter = 25 cm, and water depth = 16 cm.

We tested the rheotactic behavior of Turure and Aripo guppies (number of tested females in Table 1) in a circular-flow tank similar to the one described in Jiang et al. (2015), where guppies could swim freely in either direction: against (upstream) or with (downstream) the current (Figure 2). Two pumps were used to generate flow and were located outside the testing tank to prevent fish from hiding behind the pump. The tank was placed in a room without windows and with a fixed source of light directly above the tank.

### 2.4. Rheotaxis Trials

Each fish was individually tested in the circular-flow tank. After an acclimation period of 15 min, the pumps were turned on for 5 min to create a continuous flow. During these 5 min, the fish movement was videotaped with a webcam (Logitech C270) for later analyses. At the end of the experiment, the pumps were turned off and the fish was allowed to recover for 5 min before being anesthetized for the measurement of length (cm ± 0.001) and mass (g ± 0.0001). Even though female guppies showed no attraction to conspecific cues in an experimental flow chamber in a previous study [38], we ran a carbon filter to filter chemicals for 5 min between trials. We also recorded water temperature for each trial.

### 2.5. Video Analysis

Each individual video trial was converted to 450 images (1.5 frames per second) using the software Adapter version 2.1.6. We then used ImageJ [39] with the MtrackJ plugin [40] to track the anterior and posterior ends of each fish. The anterior and posterior end were then translated into x and y coordinates for each frame. From these coordinates, we quantified net displacement, cumulative upstream movement, flow regime experienced, and upstream orientation [41].

Net displacement was calculated as the total distance traveled during the trial: from a fish starting point, any movement in the upstream direction (against the flow) was summed up, and any movement in the downstream direction (with the flow) was subtracted, until the end point. This distance can be positive (the fish swam mostly in the upstream direction) or negative (the fish swam mostly in the downstream direction). To facilitate this inference, we also estimated the distance covered in the downstream direction in the absence of positive rheotaxis. This estimate was made using an inanimate prop (a miniature spoon with tape wrapped around the anterior tip of it) having the same mass as a guppy (0.40 g). Net downstream displacement for this mimic of a non-swimming guppy was −4146 ± 2361 cm, depending on the flow regime occupied. Thus, net displacement of a guppy less than this distance would reflect positive rheotaxis. Cumulative upstream movement was calculated as the total distance that the fish swam in the upstream direction (against the current). For these two variables, higher values indicate stronger positive rheotactic behavior. Importantly, however, a fish can show net displacement downstream despite strong positive rheotaxis. That is, in a strong current, a fish could be displaced downstream despite furiously and continuously swimming against the current. 

The flow regime experienced by the fish was determined in each frame as the presence of the fish in one of the four flow zones (Figure 2) as 0, 1, 2, or 3 for the minimal (4.2 cm/s), low (6.9 cm/s), medium (8.8 cm/s), and high (17.1 cm/s) flow zones respectively. Flow was measured by dropping food coloring in water and recording its diffusion in subsequent time frames. The 450 flow regime measurements per trial per fish were then averaged for each fish to obtain the mean flow regime score over the entire trial. A fish could either decide to avoid the flow by staying in the minimal flow zone or decide to actively swim in higher flow zones.

For upstream orientation, we determined in each of the 450 frames to what extent each fish was facing upstream by measuring the angle between the fish and the tangent of the flow. We then calculated the proportion of time the fish was aligned upstream within ±45° of the flow during the entire trial. High values for upstream orientation indicate more positive rheotactic behavior.

### 2.6. Statistical Analyses

All analyses were performed using the R language [42,43]. Significance was set at α = 0.05 and means ± standard deviations are reported throughout, unless otherwise specified. We excluded two outliers from the analysis: both from the Aripo River, one from the Below pool type and one from the Between pool type. Both fish displayed values for net displacement that were either 4 times or 15 times higher than the mean. For each of the four response variables (net displacement, cumulative upstream movement, flow regime, and upstream orientation) we used a separate linear model. We set as fixed effects, the pool type from which the fish (for F0s) or its parents (for F1s) had been collected (three levels: “Above”, “Between”, “Below”), the river (two levels: “Aripo” and “Turure”), and the interaction between pool type and river. We set as covariates, the fish mass, the mean temperature during the trial, and the fish generation (F0 or F1). For every response variable, we built a full model, and then ran a model selection procedure using Akaike Information Criterion (AIC; see supporting information for the full model selection), dropping effects that did not improve the model in the following order: mass, generation and mean temperature. 

The final model for net displacement was Y ~ MeanTemperature + pool + river + pool × river. The final model for cumulative upstream movement was Y ~ mass + MeanTemperature + generation + pool + river + pool × river. The final model for flow regime was Y ~ generation + MeanTemperature + pool + river + pool × river. The final model for upstream orientation was Y ~ pool + river + pool × river. We then checked for independence and homogeneity of the residuals and transformed the response variable if needed. Flow regime and cumulative upstream movement were log transformed to meet normality of the residuals and homoscedasticity of the variance. When pool type, or the interaction between pool type and river was significant, we explored two a priori planned contrasts [44]: Above pool type versus the combination of Between/Below (upstream vs. downstream; contrast 1); and Between versus Below (contrast 2; Figure S1). These contrasts were set up to test if there was a difference between upstream and downstream populations, but also to compare fine scale variation. Fish collected from Between pools could express high positive rheotaxis because they were located above a waterfall, but they could also express low positive rheotaxis because they were located downstream of Above fish. 

Finally, we used a generalized additive mixed model (GAMM) in the R package gamm4 [45] to analyze the temporal fish response relative to the flow (flow regime and upstream alignment) during the trial. Time was entered as frame number. We included River and Pool type as linear factors and time-by-river as a non-linear factor-smooth interaction, with the smoothing parameter estimation “REML”. Fish ID was entered as a random factor, with a random slope and a random intercept. We used a quasipoisson distribution for flow regime, and a binomial distribution for alignment (0 is not aligned, 1 is aligned). Neither GAMM model provided a good fit to the raw data. Therefore, we used generalized additive models (GAMs) in the R package mgcv [46] on the average flow regime or the average alignment per pool type and per river using a gaussian family. We recognize that, in doing so, we lost the ability to take into account individual level variation by using the mean in our model, but this was our only option. We corrected for autocorrelation of the temporal data points in our models using the corARMA function in the R package nlme [47].

## 3. Results

We first summarize our main findings. (i) Fish in both rivers showed strong positive rheotaxis. (ii) Turure and Aripo fish achieved this positive rheotaxis in different ways: fish from Turure first occupied low-flow areas, and then moved to high-flow areas, whereas fish from Aripo occupied intermediate-flow areas throughout the trial. (iii) Rheotactic behavior within each river did not differ between fish from above the upstream waterfall and those from below the downstream waterfall. 

### 3.1. Summary Statistics

Average mass of the fish used in the experiment was 0.30 ± 0.11 g, with no differences between rivers (*F_1,63_* = 0.999, *P* = 0.321), nor among the three pool types (*F_3,63_* = 0.192, *P* = 0.901). However, F0 fish were heavier than F1 fish (0.34 ± 0.08 g vs. 0.15 ± 0.07 g; *F_1,56_* = 60.6, *P* < 0.001). Average temperature during the trials was 23 ± 1 °C, with no difference for fish from different rivers (*F_3,64_* = 0.5, *P* = 0.477) or pool types (*F_3,64_* = 0.1, *P* = 0.973). Overall, fish expressed strong positive rheotactic behavior, given that net downstream displacement ranged from -1268 to 559 cm, whereas downstream displacement in the absence of swimming ranged from −6236 to −1472 cm. Fish spent 70 ± 11% of their time aligned against the flow, although this was often in low-flow zones (almost 75% of the alignment against the flow occurred in the minimal or low-flow zones). 

### 3.2. Linear Models for Rheotactic Behavior 

**Figure 3:**
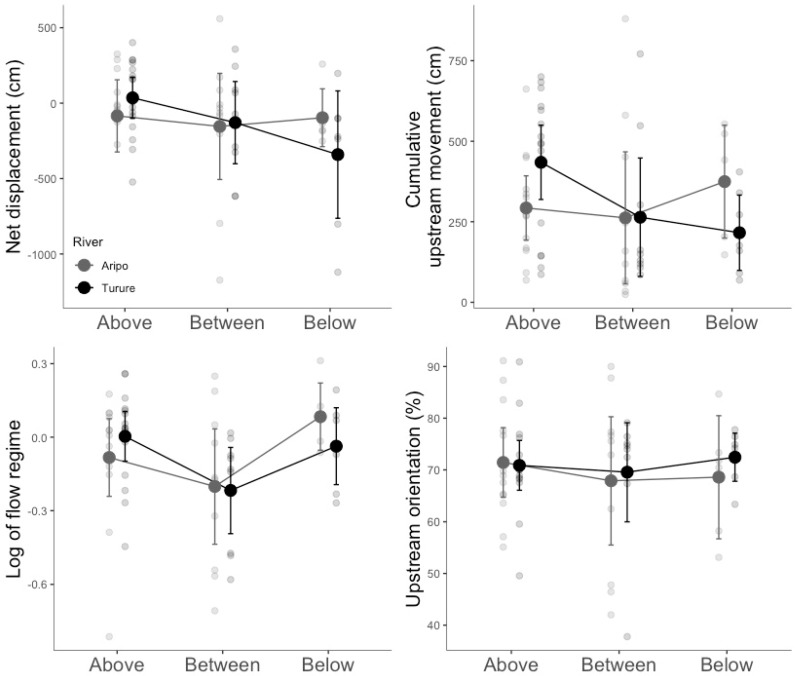
Means and 95% confidence intervals for net displacement, log of flow regime, cumulative upstream movement and upstream orientation, for each pool type in each river. The raw data points are presented in grey.

We did not detect any difference between the two rivers for the four measured variables (net displacement, cumulative upstream movement, flow regime, and upstream orientation; Table 2; Figure 3), thus indicating similar overall rheotaxis between the Turure fish and the Aripo fish. Fish from different pool types along the river gradient also expressed similar rheotactic behaviors as no differences were found among Above, Between or Below fish (Table 2; Figure 3). However, although non-significant, net displacement and cumulative upstream movement decreased from the Above to Below pool types for the Turure fish (Figure 3).

### 3.3. Generalized Additive Model for Temporal Patterns of Flow Regime and Alignment 

**Figure 4:**
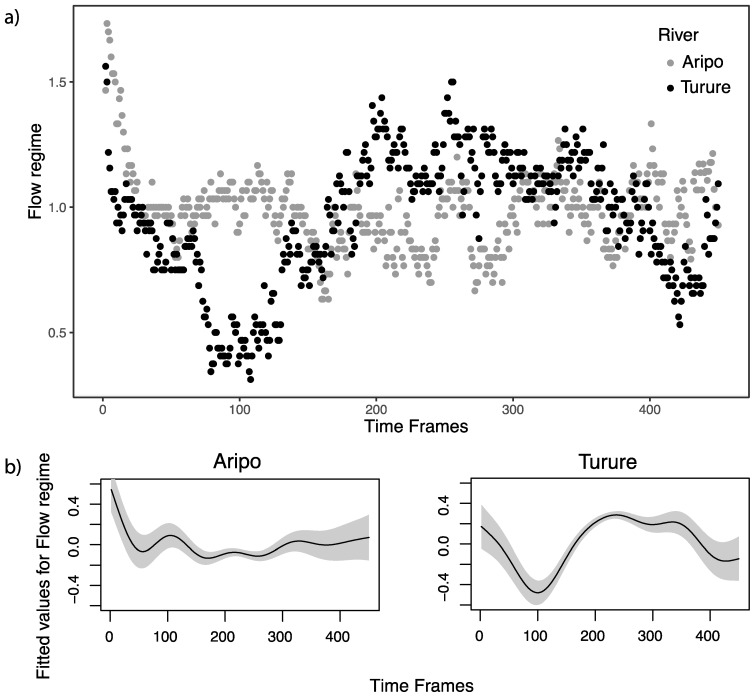
Values for flow regime during the entire duration of the trial (450 time frames over 5 min). (**a**) Individual values averaged at each time frame for each river across all pool types show a different behavioral pattern. High values mean that fish are swimming in higher flow zones. (**b**) Fitted values with GAM for the two rivers, the grey areas represent 95% confidence bands. Values are centered around 0.

Fish from the two rivers showed complex and distinct patterns of movement over the course of the trial. The Turure fish immediately positioned themselves in the low-flow zone at the beginning of the trial before swimming in higher flow zones, whereas Aripo fish maintained their position in an intermediate-flow regime during the entire trial (Figure 4; Figure S2). This difference was significant when river was included as a fixed factor in the GAM (*F* = 110.3, *P* < 2 × 10^−16^).

**Figure 5:**
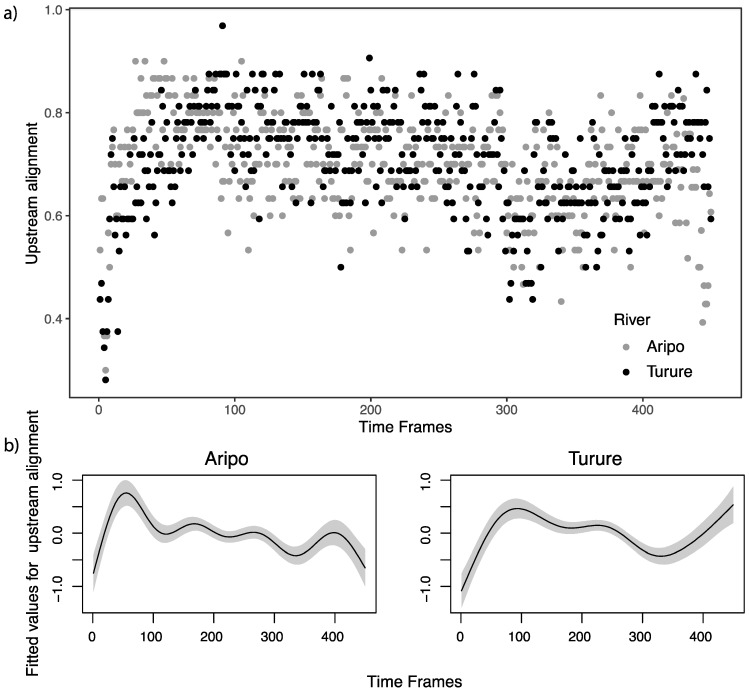
Values for alignment of the fish against the flow during the entire duration of the trial (450 time frames = 5 min). (**a**) Raw values for the two rivers. Maximum value (1) means that all fish were aligned with the flow, minimum value (0) means that none of the fish were aligned with the flow for that time frame. (**b**) Fitted values with GAM for the two rivers, the grey areas represent 95% confidence bands. Values are centered around 0.

Fish from the two rivers aligned themselves against the flow as soon as the pumps were turned on (Figure 5; Figure S3), but upstream alignment for guppies from Aripo decreased with time, whereas upstream alignment for guppies from Turure increased with time. Again, this difference was significant (*F* = 4.933, *P* = 0.026).

## 4. Discussion

### 4.1. Objective 1. Rheotaxis in Two Asymmetrically Isolated Guppy Populations

Guppies in both populations exhibited strong positive rheotaxis: they all aligned against the flow during the duration of the trial and they all swam in the upstream direction during most of the trial. The overall average tendency was still toward some net downstream displacement; yet based on comparisons with an inanimate prop, this displacement was on average 41 times less than expected if the guppies had not actively aligned against the current. Hence, our results confirm that guppies located in the upstream reaches of rivers express strong positive rheotaxis: that is, a strong tendency to swim against the flow [48]. 

Of additional interest, strong positive rheotaxis in the Turure population might reflect contemporary adaptive evolution following their introduction. We feel this argument is justified given several previous observations: rheotaxis has a strong genetic basis in various fishes [49,50] and many Turure guppies were clearly displaced downstream soon after their introduction [6,15,31,33]. This second assertion is supported by the fact that the genotypic signature of introduced guppies replaced many native genotypes up to at least 1 km downstream of the introduction site in the Turure [6,15,31,33]. Given that the introduced Turure population initially must have shown substantial downstream gene flow, strong positive rheotaxis in the present-day population would suggest an evolutionary change following introduction. However, ambiguity remains for this inference of contemporary (rapid) evolution. One reason is that the source population for the Turure introduction is not certain and so we could not quantify rheotaxis in the specific ancestral population. We therefore instead focused on exploring whether the introduced guppies now showed similar positive rheotaxis to another upstream population. As a result, we cannot be certain how much, and in which direction, the Turure population has changed in their rheotactic behavior. Yet we do know that the source population was at least not from an isolated location above barrier waterfalls [29]. Thus, as seen in numerous other fishes (review in [22]), the downstream origin of the Turure fish was likely to dictate initially weak positive rheotaxis, a supposition supported by the dramatic effect their downstream movement had on native populations [15,31,33]. Further support comes from Mohammed et al. (2012), who found that guppies located in lower reaches of a stream were more likely to be swept downstream than guppies located further upstream [48]. It therefore seems likely that the current strong positive rheotaxis in the Turure River reflects at least some (and perhaps substantial) contemporary evolution following their introduction more than 60 years ago.

### 4.2. Objective 2. Multiple Solutions to the Same Problem

Guppies in the two populations achieved the same overall performance (positive rheotaxis) using two different behavioral strategies, most notably in their temporal patterns of occupying flow zones (Figure 4). Aripo guppies generally occupied an intermediate-flow zone throughout the trials. Turure guppies, by contrast, initially all occupied a lower flow zone than all Aripo fish. After about two minutes (200 time frames), Turure guppies nearly all occupied a higher flow zone than nearly all Aripo fish. Only after around three and a half minutes (320 time frames) did Turure guppies converge on a similar flow regime to Aripo guppies. These dramatically different temporal responses of guppies from the two populations suggests the evolution of very different strategies for coping with water flow: that is, multiple behavioral solutions have been used to the same evolutionary problem—as has been suggested for such many-to-one trait-to-performance mapping situations. Overall, this evidence of populations evolving different solutions to a similar overall selective problem is consistent with recent work on other traits in guppies [51,52,53], other fishes (e.g., [25]), and other organisms in general [54].

We do not know the specific reason why the two populations now show such different behavioral solutions (temporal differences in flow zone choice and in upstream orientation) to achieve roughly the same performance (positive rheotaxis and similar net displacement). Nevertheless, we here speculate in hopes of generating hypotheses that might inspire future hypothesis testing. Some reasons might reflect contingencies associated with the specific pool of fish introduced, such as the river topology of the source or other environmental particularities. Alternatively, the differences between populations could be explained by recent selective forces; that is, site-specific contemporary evolution of the Turure fish following their introduction. The portion of the Turure River where guppies were introduced in 1957 is locally called the “Turure water steps” and consists of high limestone waterfalls each over 7 m in height. This considerable drop of water creates refuge zones at the bottom of the waterfall. We speculate that, in case of a flood or other increase in flow, guppies are probably able to use these low-flow zones to hide. These refuge zones are not as obvious in the Aripo population, where the waterfalls are much smaller. Perhaps this difference shapes the differential behavior solutions of the two populations. Alternatively, perhaps the young Turure population will eventually converge on the solution achieved by the much older Aripo population. 

### 4.3. Objective 3. Rheotaxis along A River Gradient

Guppies sampled in the different pool types along each river expressed similar overall rheotactic behavior. This similarity among pools located on either side of waterfalls could be due to several reasons. First, guppies (and especially females) express site fidelity and do not migrate seasonally for reproduction or for food. By contrast, the majority of studies on rheotaxis have focused on salmonids, which are typically migratory below (but not above) waterfalls, potentially favoring greater divergence in rheotaxis across such barriers than would be the case for non-migratory guppies. Second, the scale of our study might have been too small to detect any difference in rheotactic behavior: the sampled pools were only separated by 75 m for Aripo and 85 m for Turure, which is a scale on which downstream gene flow can be very high [33,55,56], potentially homogenizing adaptive variation. Furthermore, even though guppies sampled in the Turure were the most upstream site, this was not the case for the Aripo, which could also receive migrants from upstream. Third, waterfalls in the Turure and Aripo might not have been high enough to prevent upstream migration, instead allowing mixing of the upstream and downstream populations under at least some conditions. Although this last explanation is perhaps unlikely, it would be more likely for the Aripo River where the waterfalls are much smaller than in the Turure. Another potential explanation for the lack of small-scale differences is that guppies from these two rivers were all sampled from pools located in low predation areas, meaning that any emigrating fish from above the waterfall to the below pool would not be as disadvantaged as if they were emigrating into a high predation environment (see [17]).

### 4.4. But Is It Evolution?

Our experiments used a mixture of wild-captured and first-generation lab reared guppies, which might therefore have retained plastic or maternal contributions to rheotactic behavior. Thus, technically, we cannot state with absolute confidence the genetic contribution to the patterns and differences observed. Nevertheless, the lab-reared guppies never experienced a water current before the experiment, and the wild-caught guppies had not experienced any current for 8 months prior to testing. Combined with the previously-noted genetic basis for rheotaxis differences observed in other fishes [49,50], we therefore suspect that at least some of the differences in temporal patterns do reflect genetic differences. Moreover, we know from previous studies that rheotaxis is a behavior mediated by the lateral line in fish [19,57], and that variation in lateral line morphology has been found to be associated with genetic differences in several guppy populations [58]. Formal studies investigating the genetic basis versus phenotypic plasticity of rheotaxis would be required to answer this question.

## 5. Conclusions

We provided evidence that upstream Turure and Aripo guppies demonstrate similar strong positive rheotaxis. That is, all of the guppies aligned against the flow for the majority of the trial, and they all swam in the upstream direction much more than expected had they exhibited passive responses. However, we also found a striking behavioral difference between the populations in how they achieved this level of positive rheotaxis, suggesting alternative behavioral solutions to the same functional challenge. At a smaller scale, rheotaxis across waterfalls within a river was similar, suggesting that selection for positive rheotaxis was not strong enough at that scale. Overall, our findings imply that upstream guppies have evolved different behavioral mechanisms—perhaps rapidly—to maintain populations over barrier waterfalls despite asymmetric isolation. 

## Figures and Tables

**Table 1:** Sample sizes (numbers of fish analyzed for rheotaxis) in the Aripo and Turure rivers. The unbalanced design reflects lab mortality.

Site Locations Relative to Waterfalls	Aripo		Turure	
	F0	F1	F0	F1
Above	11	2	14	2
Between	5	5	6	3
Below	6	0	5	2
**Total**	**22**	**7**	**25**	**7**

**Table 2:** Output of the linear models for each of the four response variables.

Response Variable	Adj. R^2^	*F*	*P*
**Net displacement**	0.00		
Mean temperature		0.771	0.384
River		0.882	0.352
Pool		0.152	0.860
River × Pool		1.214	0.305
**Log of cumulative upstream movement**	0.28		
Mass		0.992	0.324
Mean temperature		2.028	0.160
Generation		6.004	0.018
River		1.885	0.176
Pool		0.728	0.488
River × Pool		1.268	0.290
**Log of flow regime**	0.54		
Mean temperature		0.926	0.340
Generation		32.020	6.29 × 10^−7^
River		1.250	0.269
Pool		0.849	0.433
River × Pool		1.364	0.265
**Upstream orientation**	−0.07		
River		0.017	0.900
Pool		0.293	0.748
River × Pool		0.169	0.845

## Supplementary Materials

The following are available online at https://www.mdpi.com/2073-4425/11/2/180/s1, Planned contrasts between the three levels of pool. We first compared the upstream pool type (Above) with the downstream pool types (Between and Below), and then we compared the two downstream pool types (Between vs. Below). With these two contrasts, we were able to explore fine scale variation in rheotaxis behavior. Figure S2: Values for flow regime during the entire duration of the trial (450 time frames = 5 min), for the three pool types. The horizontal line represents the overall mean value for flow regime. Figure S3: Values for alignment during the entire duration of the trial (450 time frames = 5 min), for the three pool types. The horizontal line represents the overall mean value for alignment. Supplementary File F1: Linear model selection.
