# Supplementary material for: Asymmetric Isolation and the Evolution of Behaviors Influencing Dispersal: Rheotaxis of Guppies above Waterfalls
Source: Genes (Basel). 2020 Feb 9;11(2):180. doi: 10.3390/genes11020180 (PMC7073897; doi:10.3390/genes11020180)
Supplement: Supplementary file 1 [file genes-11-00180-s001.pdf]

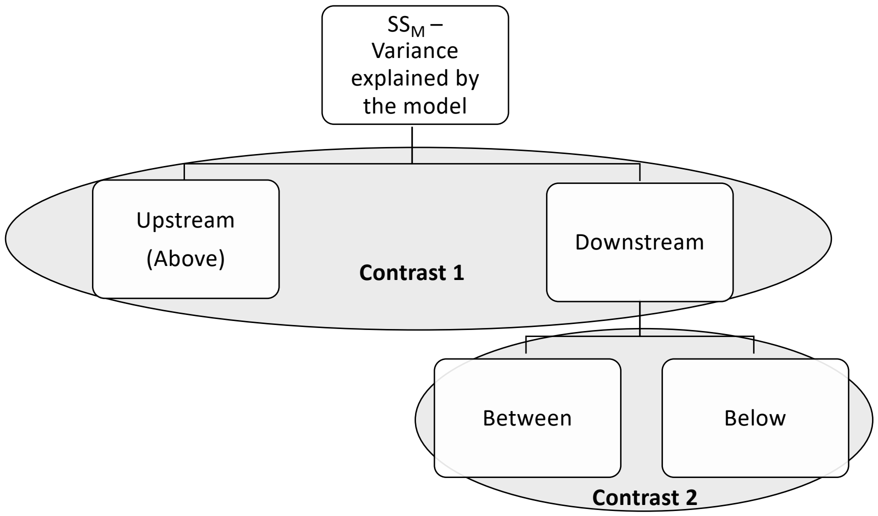

Fig S1 Planned contrasts between the 3 levels of pool. We first compared the upstream pool type (Above) with the downstream pool types (Between and Below), then we compared the two downstream pool types (Between vs. Below). With these two contrasts, we were able to explore fine scale variation in rheotaxis behavior.

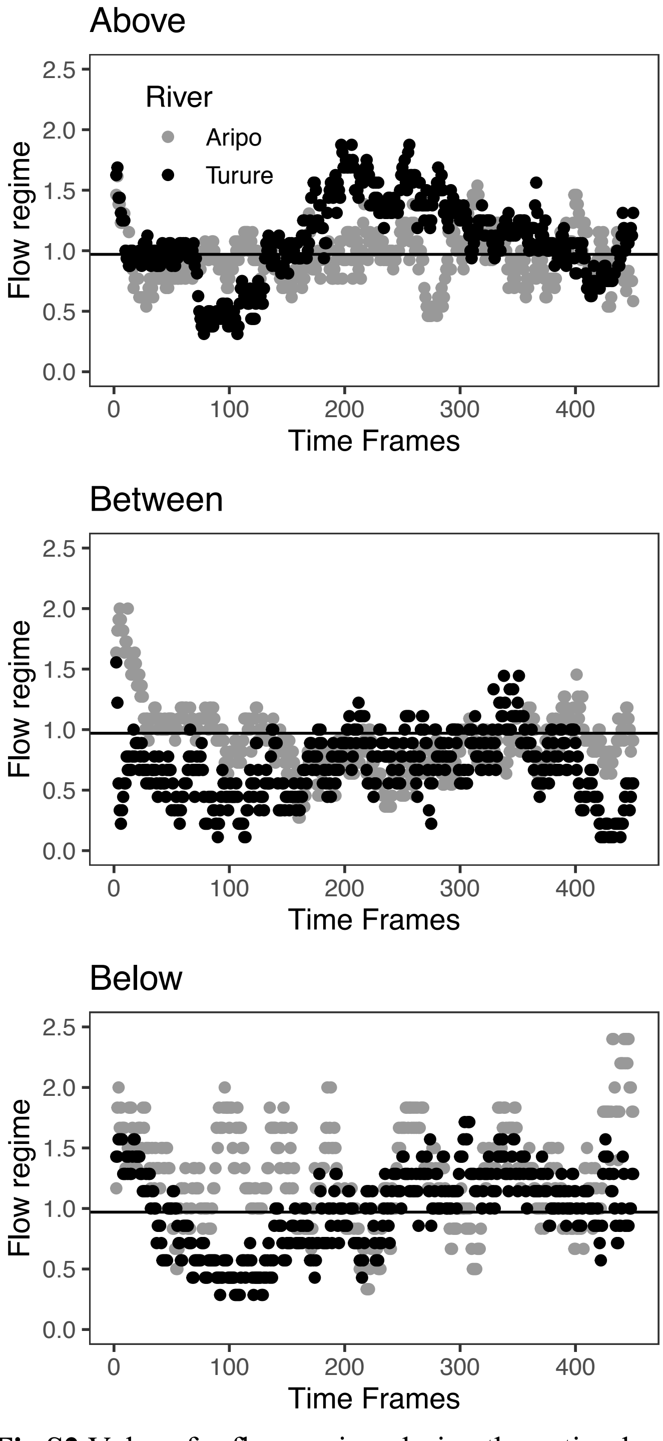

Fig S2 Values for flow regime during the entire duration of the trial (450 time frames = 5 minutes), for the three pool types. The horizontal line represents the overall mean value for flow regime.

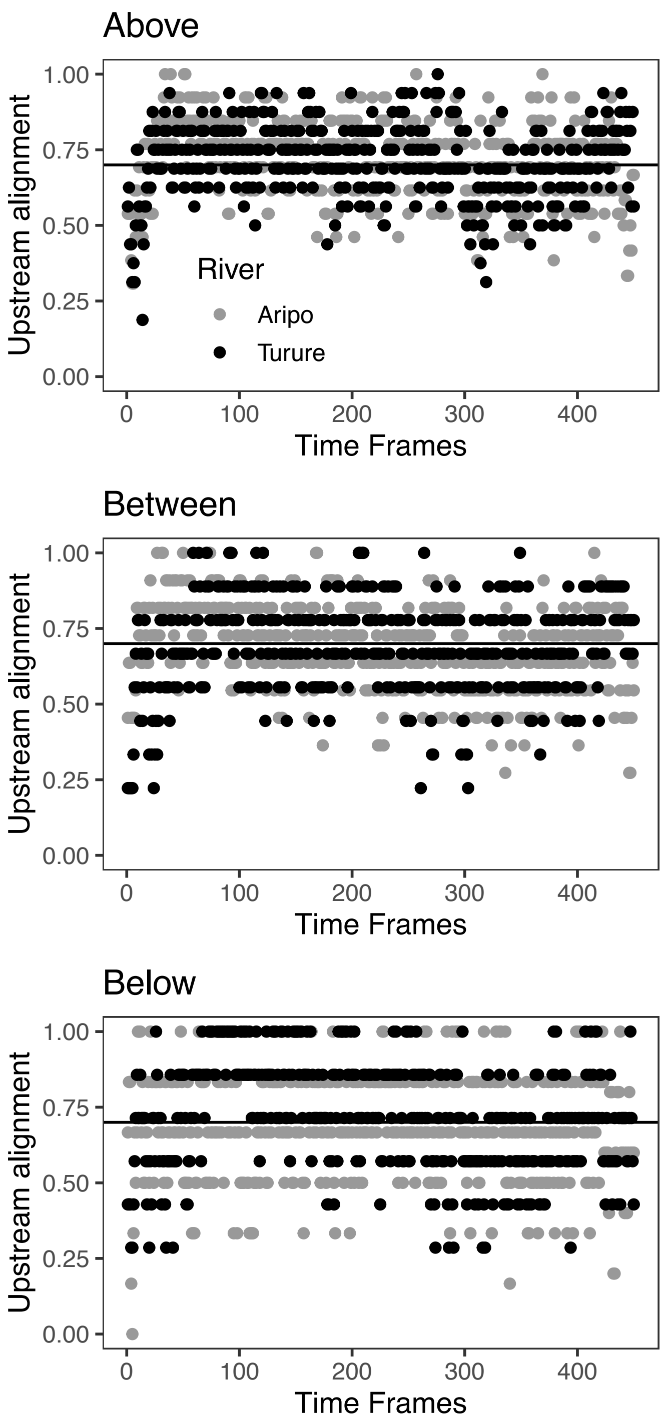

Fig S3 Values for alignment during the entire duration of the trial (450 time frames = 5 minutes), for the three pool types. The horizontal line represents the overall mean value for alignment.

Asymmetric isolation and the evolution of behaviors influencing dispersal: rheotaxis of guppies above waterfalls

Léa Blondel

November 7th, 2019

Full models

There are four response variables (all continuous) for which we built a model:

- Net displacement
- Cumulative upstream movement
- Flow regime
- Upstream orientation

The fixed effects are:

- MeanTemp - continuous variable
- Weight - continuous variable
- Pool - 3 levels: Above, Between, Below
- River- 2 levels: Aripo, Turure
- Generation - 2 levels: F0, F1

We used planned contrasts to test for specific comparisons:

- Contrast 1: Upstream (Above) vs Downstream (Between + Below)
- Contrast 2: Between vs Below

Response variable : Net displacement

The full model is: netDisplacement ~ weight + meanTemp + generation + river * pool

We test the importance of fixed effects:

```
modelDisp2 <- lm(data = rheoB,
                 netDisplacement ~ meanTemp + generation + river * pool)

modelDisp3 <- lm(data = rheoB,
                 netDisplacement ~ meanTemp + river * pool)

modelDisp4 <- lm(data = rheoB,
                 netDisplacement ~ river * pool)
```

Table 1: comparison of the AIC scores for each model for net displacement

Model	AICc
modelDispFull	909.7565
modelDisp2	907.0281
modelDisp3	905.5300
modelDisp4	903.7383

We kept model number 3 (netDisplacement ~ meanTemp + river * pool) because it does not differ from >2 with the model with the lowest AIC.

Checking the assumptions:

```
plot(modelDisp3, 1) #checking the variance: OK
```

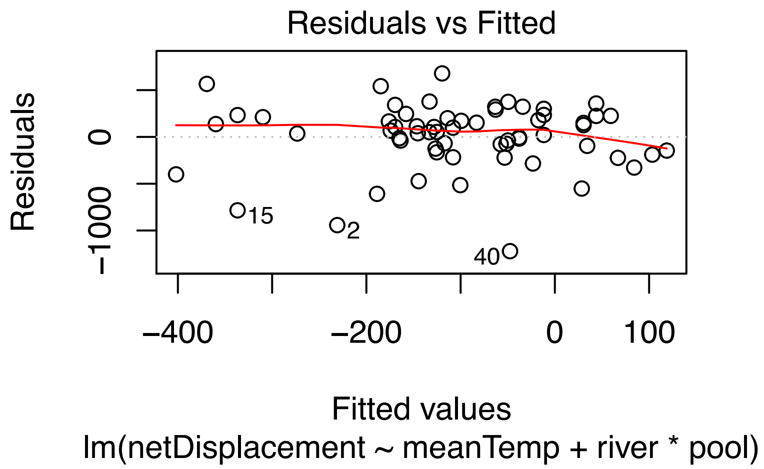


```
plot(modelDisp3, 2) #checking the normality of the residuals: OK
```

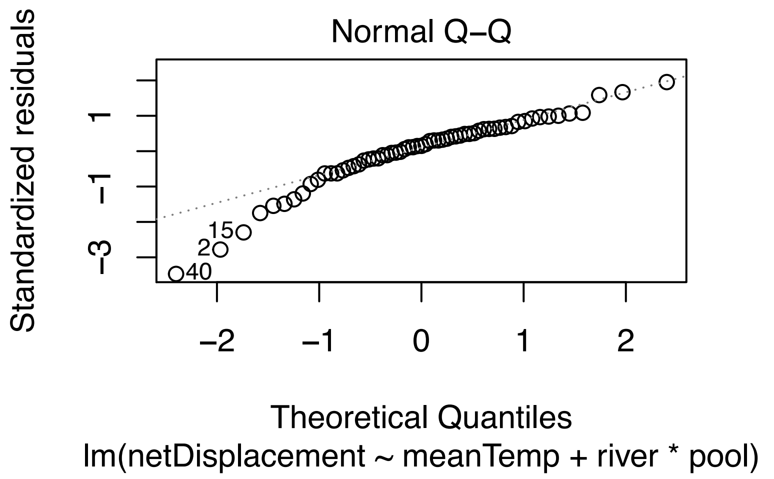

Here is our **final model for net displacement**:

```
##
## Call:
## lm(formula = netDisplacement ~ meanTemp + river * pool, data = rheoB)
##
## Residuals:
##     Min      1Q  Median      3Q     Max
## -1220.9  -146.0    52.4   224.6   678.9
##
## Coefficients:
##                         Estimate Std. Error t value Pr(>|t|)
## (Intercept)              794.784   1006.789   0.789    0.433
## meanTemp                 -38.376     43.715  -0.878    0.384
## riverTurure              129.821    138.205   0.939    0.352
## poolBelow                 -6.123    182.198  -0.034    0.973
## poolBetween              -81.649    155.747  -0.524    0.602
## riverTurure:poolBelow   -384.044    248.181  -1.547    0.128
## riverTurure:poolBetween  -77.943    219.250  -0.355    0.724
##
## Residual standard error: 368.9 on 54 degrees of freedom
## Multiple R-squared:  0.1032, Adjusted R-squared:  0.003549
## F-statistic: 1.036 on 6 and 54 DF,  p-value: 0.4127
```

```
## Anova Table (Type III tests)
##
## Response: netDisplacement
##              Sum Sq Df F value Pr(>F)
## (Intercept)   84810  1  0.6232 0.4333
## meanTemp     104876  1  0.7706 0.3839
## river        120081  1  0.8824 0.3517
## pool          41329  2  0.1518 0.8595
## river:pool   330431  2  1.2140 0.3050
## Residuals   7348878 54
```

Response variable: Cumulative Upstream Movement

```
modelUpstreamMovFull <- lm(data = rheoB,
                           cumulativeUpstreamMov ~ weight + meanTemp + generation + river * pool)
```

We test the importance of fixed effects.

```
modelUpstreamMov2 <- lm(data = rheoB,
                   cumulativeUpstreamMov ~ generation + meanTemp + river * pool)

modelUpstreamMov3 <- lm(data = rheoB,
                   cumulativeUpstreamMov ~ meanTemp + river * pool)

modelUpstreamMov4 <- lm(data = rheoB,
                   cumulativeUpstreamMov ~ river * pool)
```

Table 2: comparison of the AIC scores for each model for cumulative upstream movement

Model	AICc
modelUpstreamMovFull	834.9279
modelUpstreamMov2	833.0833
modelUpstreamMov3	833.2634
modelUpstreamMov4	836.1396

We kept the full model (cumulativeUpstreamMov ~ weight + meanTemp + meanTemp + river * pool) because it does not differ > 2 from the model with the lowest AIC.

Checking the assumptions

```
plot(modelUpstreamMovFull, 1) #checking the variance: not met
```

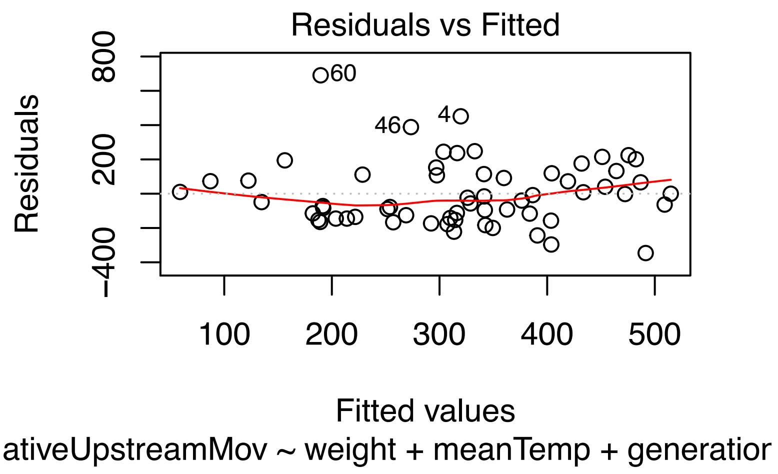

```r
plot(modelUpstreamMovFull, 2) #checking the normality: OK
```

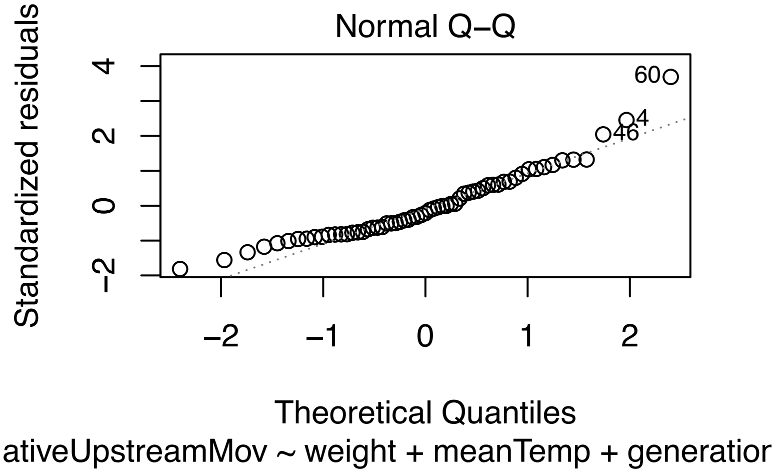

We transformed the response variable using log because the assumptions were not met.

```r
modelUpstreamMovFinal <- lm(data = rheoB,
                    log10(cumulativeUpstreamMov) ~ weight + meanTemp + generation + river * pool)
```

And check again the assumptions:

```r
plot(modelUpstreamMovFinal, 1) #checking the variance: OK
```

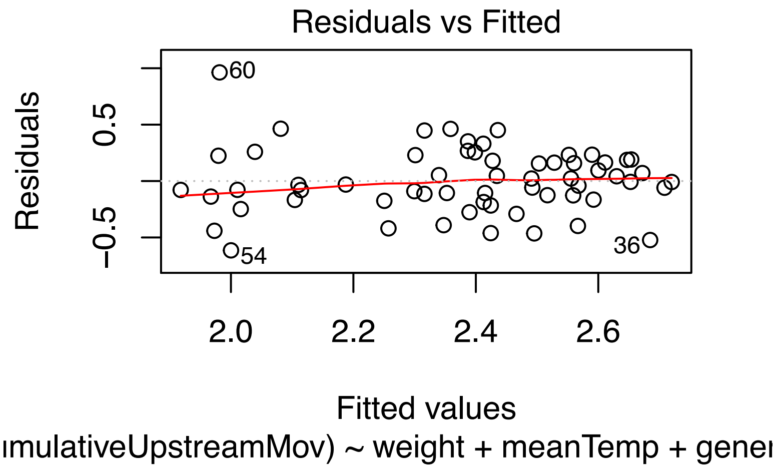


```r
plot(modelUpstreamMovFinal, 2) #checking the normality of the residuals: OK
```

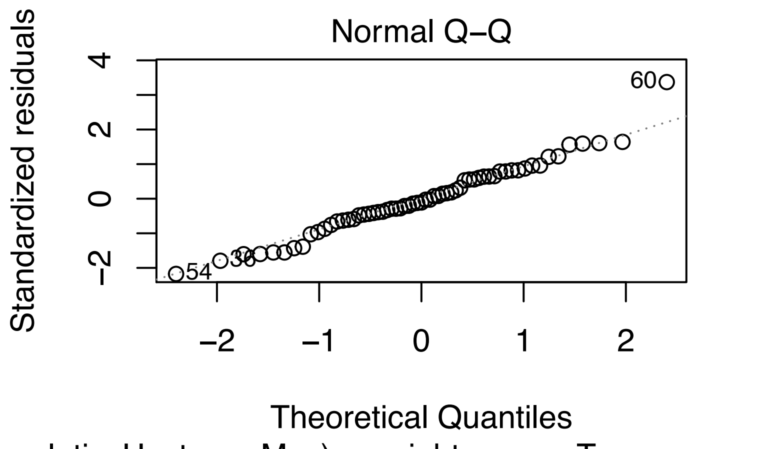

Here is our **final model for cumulative upstream movement**:

```
##
## Call:
## lm(formula = log10(cumulativeUpstreamMov) ~ weight + meanTemp +
##     generation + river * pool, data = rheoB)
##
## Residuals:
##      Min       1Q   Median       3Q      Max
## -0.61441 -0.16483 -0.03102  0.18755  0.96311
##
## Coefficients:
##                         Estimate Std. Error t value Pr(>|t|)
## (Intercept)              1.17727    1.03408   1.138   0.2601
## weight                  -0.52821    0.53037  -0.996   0.3239
## meanTemp                 0.06192    0.04347   1.424   0.1604
## generationF1            -0.39125    0.15968  -2.450   0.0177 *
## riverTurure              0.16126    0.11744   1.373   0.1756
## poolBelow                0.10292    0.15422   0.667   0.5075
## poolBetween             -0.09712    0.13601  -0.714   0.4784
## riverTurure:poolBelow   -0.33438    0.21006  -1.592   0.1175
## riverTurure:poolBetween -0.09038    0.18275  -0.495   0.6230
## ---
## Signif. codes:  0 '***' 0.001 '**' 0.01 '*' 0.05 '.' 0.1 ' ' 1
##
## Residual standard error: 0.3073 on 52 degrees of freedom
## Multiple R-squared:  0.3784, Adjusted R-squared:  0.2827
## F-statistic: 3.956 on 8 and 52 DF,  p-value: 0.001022
```

```
## Anova Table (Type III tests)
##
## Response: log10(cumulativeUpstreamMov)
##             Sum Sq Df F value  Pr(>F)
## (Intercept) 0.1224  1  1.2961 0.26014
## weight      0.0937  1  0.9919 0.32390
```

```
## meanTemp    0.1915  1  2.0283 0.16037
## generation  0.5669  1  6.0037 0.01768 *
## river       0.1780  1  1.8854 0.17561
## pool        0.1374  2  0.7276 0.48793
## river:pool  0.2394  2  1.2675 0.29008
## Residuals   4.9105 52
## ---
## Signif. codes:  0 '***' 0.001 '**' 0.01 '*' 0.05 '.' 0.1 ' ' 1
```

Response variable: Flow regime

```
modelFlowFull <- lm(data = rheoB,
                    flowRegime ~ weight + generation + meanTemp + river * pool)
```

We now test the importance of fixed effects.

```
#Now chosing the best fixed effects
modelFlow2 <- lm(data = rheoB,
                 flowRegime ~ generation + meanTemp + river * pool)

modelFlow3 <- lm(data = rheoB,
                 flowRegime ~ meanTemp + river * pool)

modelFlow4 <- lm(data = rheoB,
                 flowRegime ~ river * pool)
```

Table 3: comparison of the AIC scores for each model

Model	AICc
modelFlowFull	50.74932
modelFlow2	48.20284
modelFlow3	63.26690
modelFlow4	73.83203

We kept model number 2 (flowRegime ~ generation + meanTemp + river * pool) because it has the lowest AIC and differs from > 2 from the other models.

We can check the assumptions

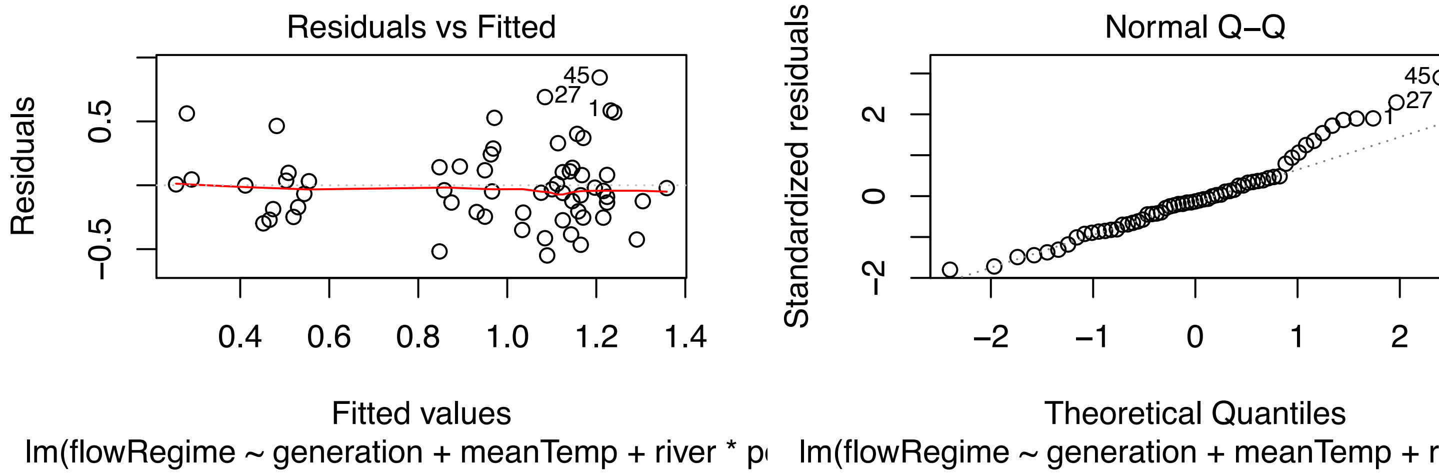

The variance and the normality of the results don't seem to meet the assumptions.

We transform the data to meet the assumtpions of normality and homoscedasticity using log.

```
modelFlowFinal <- lm(data = rheoB,
                   log10(flowRegime) ~  generation + meanTemp + river * pool)
```

We check again the assumptions

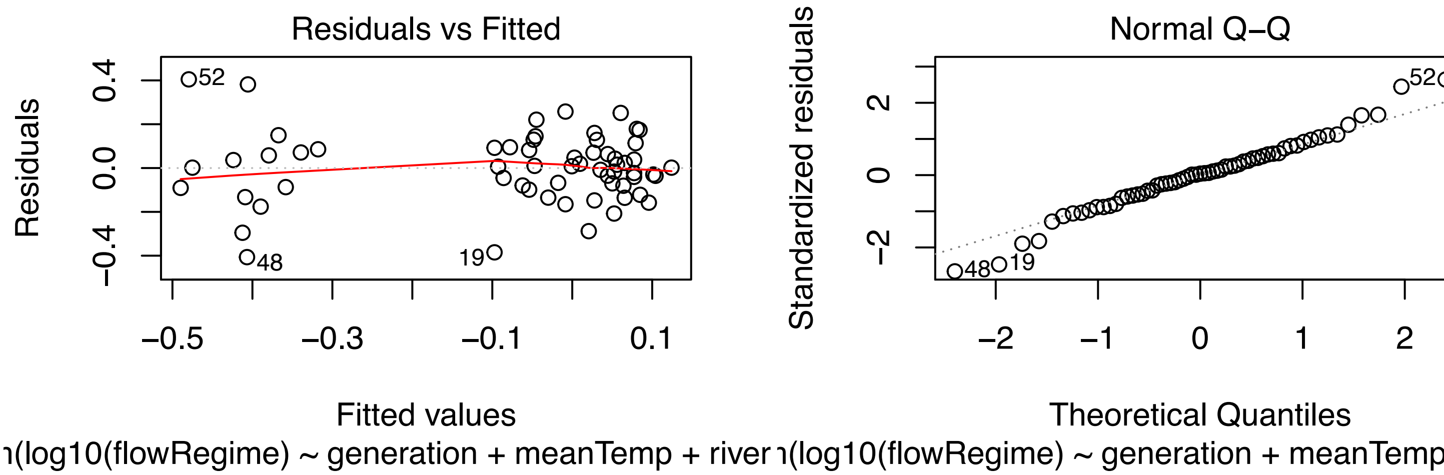

Here is **our final model for Flow regime**:

```
##
## Call:
## lm(formula = log10(flowRegime) ~ generation + meanTemp + river *
##     pool, data = rheoB)
##
## Residuals:
```

```
##      Min       1Q   Median       3Q      Max
## -0.40663 -0.08710  0.00629  0.08557  0.40481
##
## Coefficients:
##                         Estimate Std. Error t value Pr(>|t|)
## (Intercept)             -0.54609    0.54737  -0.998    0.323
## generationF1            -0.36760    0.06496  -5.659 6.29e-07 ***
## meanTemp                 0.02268    0.02357   0.962    0.340
## riverTurure              0.06999    0.06260   1.118    0.269
## poolBelow                0.10657    0.08290   1.286    0.204
## poolBetween              0.01593    0.07293   0.218    0.828
## riverTurure:poolBelow   -0.07973    0.11325  -0.704    0.485
## riverTurure:poolBetween -0.16325    0.09934  -1.643    0.106
## ---
## Signif. codes:  0 '***' 0.001 '**' 0.01 '*' 0.05 '.' 0.1 ' ' 1
##
## Residual standard error: 0.1671 on 53 degrees of freedom
## Multiple R-squared:  0.5912, Adjusted R-squared:  0.5372
## F-statistic: 10.95 on 7 and 53 DF,  p-value: 1.856e-08
```

```
## Anova Table (Type III tests)
##
## Response: log10(flowRegime)
##              Sum Sq Df F value    Pr(>F)
## (Intercept) 0.02778  1  0.9953    0.3230
## generation  0.89362  1 32.0199 6.289e-07 ***
## meanTemp    0.02585  1  0.9264    0.3402
## river       0.03489  1  1.2500    0.2686
## pool        0.04741  2  0.8494    0.4334
## river:pool  0.07612  2  1.3638    0.2645
## Residuals   1.47914 53
## ---
## Signif. codes:  0 '***' 0.001 '**' 0.01 '*' 0.05 '.' 0.1 ' ' 1
```

Response variable : Upstream orientation

```
modelOrientationFull <- lm(data = rheoB,
                    upstreamOrientation ~ weight + generation + meanTemp + river * pool)
```

We test the importance of the fixed effects:

```
modelOrientation2 <- lm(data = rheoB,
                 upstreamOrientation ~ generation + meanTemp + river * pool)

modelOrientation3 <- lm(data = rheoB,
                 upstreamOrientation ~ meanTemp + river * pool)

modelOrientation4 <- lm(data = rheoB,
                  upstreamOrientation ~ river * pool)
```

Table 4: comparison of the AIC scores for each model

Model	AICc
modelOrientationFull	488.2430
modelOrientation2	487.3616
modelOrientation3	484.8970
modelOrientation4	482.2850

We kept model number 4 (upstreamOrientation ~ river * pool) because it has the lowest AIC and differs from > 2 from the other models.

Checking assumptions

```
#Checking assumptions
plot(modelOrientation4, 1) #checking the variance: OK
```

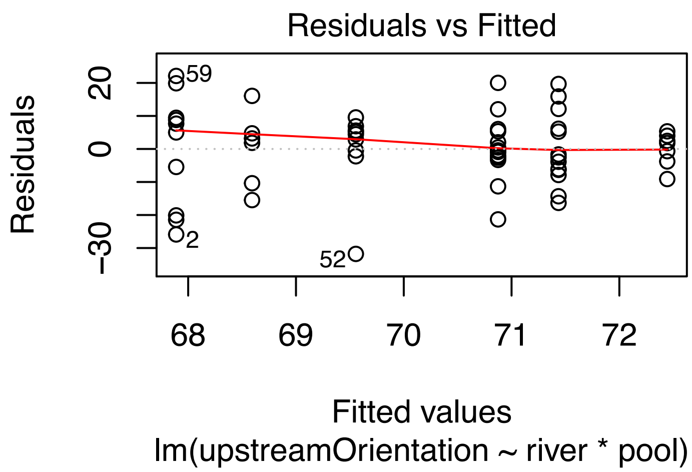


```
plot(modelOrientation4, 2) #checking the normality: OK
```

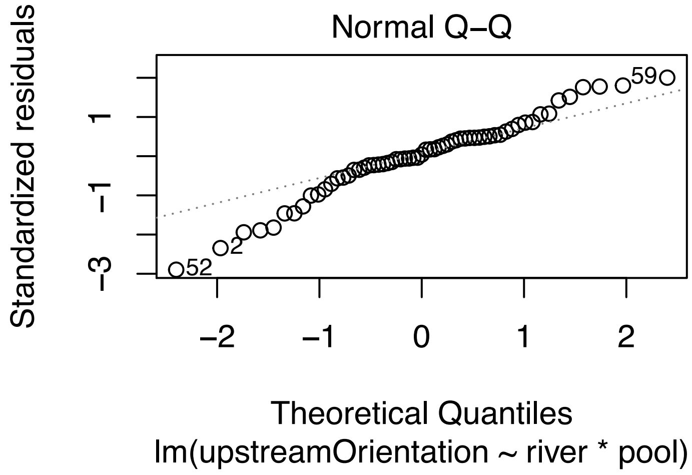

Here is our **final model for upstream orientation**:

```
summary(modelOrientation4)
```

```
##
## Call:
## lm(formula = upstreamOrientation ~ river * pool, data = rheoB)
##
## Residuals:
##     Min      1Q  Median      3Q     Max
## -31.778  -3.880   0.458   5.556  22.111
##
## Coefficients:
##                         Estimate Std. Error t value Pr(>|t|)
## (Intercept)              71.4359     3.2266  22.139   <2e-16 ***
## riverTurure              -0.5609     4.3440  -0.129    0.898
## poolBelow                -2.8433     5.7418  -0.495    0.622
## poolBetween              -3.5470     4.8934  -0.725    0.472
## riverTurure:poolBelow     4.4127     7.7950   0.566    0.574
## riverTurure:poolBetween   2.2276     6.8879   0.323    0.748
## ---
## Signif. codes:  0 '***' 0.001 '**' 0.01 '*' 0.05 '.' 0.1 ' ' 1
##
## Residual standard error: 11.63 on 55 degrees of freedom
## Multiple R-squared:  0.01779,    Adjusted R-squared:  -0.07151
## F-statistic: 0.1992 on 5 and 55 DF,  p-value: 0.9615
```

```
Anova(modelOrientation4, type = "III")
```

```
## Anova Table (Type III tests)
##
## Response: upstreamOrientation
##             Sum Sq Df  F value Pr(>F)
## (Intercept)  66340  1 490.1574 <2e-16 ***
```

```
## river            2  1   0.0167 0.8977
## pool            79  2   0.2925 0.7476
## river:pool      46  2   0.1691 0.8449
## Residuals     7444 55
## ---
## Signif. codes:  0 '***' 0.001 '**' 0.01 '*' 0.05 '.' 0.1 ' ' 1
```
